# Antibiotic Use as a Tragedy of the Commons: A Cross-Sectional Survey

**DOI:** 10.1155/2014/837929

**Published:** 2014-01-22

**Authors:** Kieran S. O'Brien, Seth Blumberg, Wayne T. A. Enanoria, Sarah Ackley, Nicolas Sippl-Swezey, Thomas M. Lietman

**Affiliations:** ^1^F.I. Proctor Foundation, University of California, San Francisco, CA 94143-0412, USA; ^2^Fogarty International Center, National Institutes of Health, Bethesda, MD 20892-2220, USA; ^3^Department of Ophthalmology, University of California, San Francisco, CA 94143-0730, USA; ^4^Department of Epidemiology & Biostatistics, University of California, San Francisco, CA 94107-0560, USA

## Abstract

*Background*. Many believe antibiotic use results in a tragedy of the commons, since overuse may lead to antibiotic resistance and limiting use would benefit society. In contrast, mass antibiotic treatment programs are thought to result in community-wide benefits. A survey was conducted to learn the views of infectious disease experts on the individual- and societal-level consequences of antibiotic use. *Methods*. The survey instrument was designed to elicit opinions on antibiotic use and resistance. It was sent via SurveyMonkey to infectious disease professionals identified through literature searches. Descriptive statistics were used to analyze the data. *Results*. A total of 1,530 responses were received for a response rate of 9.9%. Nearly all participants believed antibiotic use could result in a tragedy of the commons, at least in certain circumstances (96.0%). Most participants did not believe mass antibiotic treatment programs could produce societal benefits in an antibiotic-free society (91.4%) or in the United States (94.2%), though more believed such programs would benefit antibiotic-free societies compared to the United States (*P* < 0.001). *Conclusions*. The experts surveyed believe that antibiotic use can result in a tragedy of the commons and do not believe that mass treatment programs benefit individuals or society.

## 1. Introduction

A tragedy of the commons occurs when multiple individuals, acting solely out of self-interest, ultimately exhaust a limited shared resource despite the fact that it is not in the community's long-term interests [1, 2]. Antibiotic use has been called a tragedy of the commons, because although individuals might benefit from the use of antibiotics, concerns exist about the irreparable societal effects of antibiotic resistance developing from the overuse and misuse of antibiotics in clinical and agricultural settings [3–5]. Limiting the use of antibiotics is recommended to reduce antibiotic resistance [6, 7]. Many hospitals have implemented antibiotic stewardship programs to limit the overuse and misuse of antibiotics, in part to prevent the detrimental societal-level consequences that result from a loss of antibiotic effectiveness [8, 9]. In fact, several countries have even launched national programs to reduce unnecessary antibiotic use [10]. Despite the widespread public perception that antibiotic overuse results in a tragedy of the commons, mathematical models are rarely able to provide evidence for this or define the scenarios required for it to occur [11–15].

At the same time, mass antibiotic treatment programs have been proposed to reduce morbidity and mortality from infectious diseases in certain communities. For example, the World Health Organization endorses repeated nonspecific, mass antibiotic treatment as a key component of trachoma elimination programs [16, 17]. Such programs have been shown to benefit not only those treated with antibiotics, but untreated individuals in the community as well, resulting in reductions in disease at both the individual and societal levels [18, 19]. Moreover, these programs may impact a wide range of morbidities including malaria, respiratory infections, and diarrheal diseases [20–24]. Mass antibiotic treatment programs have even been shown to reduce overall mortality and are being proposed with these broader aims [25, 26].

Such programs have thus been shown to benefit both individuals and the larger society. Yet, concerns about antibiotic resistance, including resistance potentially caused by mass treatment, indicate that there may be a point at which individual and public health interests cross. Understanding when and how this point might occur is an important goal, but mathematical models have yet to clearly define this threshold [11]. In light of these dissonant viewpoints, the objective was to survey infectious disease professionals to understand their beliefs about the consequences of antibiotic use at both the individual and community levels.

## 2. Methods

In September 2012, a cross-sectional survey was sent to professionals with expertise in infectious diseases and antibiotic resistance. In order to survey a broad cross-section of such experts, PubMed and Web of Science were searched for articles published on antibiotic resistance and infectious diseases from 2008 to 2012. Emails of corresponding authors were extracted, with duplicates removed. Similarly, infectious disease modelers were targeted by extracting emails of authors who published in one of five journals focused solely on mathematical modeling (Journal of Mathematical Biology, Journal of Theoretical Biology, Bulletin of Mathematical Biology, Mathematical Biosciences, and Epidemics) on infectious diseases during the same time period. The authors who were identified in the searches and who identified themselves as working in infectious diseases were deemed eligible. Those with invalid email addresses and who had previously opted out of receiving emails from SurveyMonkey were ineligible. The eligibility of nonresponders was difficult to determine with the broad, electronic approach to sampling, but the authors received numerous direct email messages from both responders and nonresponders. As some of these messages indicated ineligibility for survey participation, these messages were tracked and categorized to help provide some inference about the eligibility of nonresponders.

The 18-item survey consisted of Likert items and multiple choice questions designed to elicit opinions on the individual- and societal-level consequences of antibiotic use and resistance (all questions included in Tables 1 and 2). The questions were developed with consideration of previous surveys that were conducted to elicit physicians' views on the causes of antibiotic resistance [27–30]. The questions were designed to complement previous work in this area by expanding the surveyed population to include those working in multiple related disciplines and to elicit perceptions on mass treatment programs and antibiotic use as a tragedy of the commons. Several Likert statements were adapted from a previous study [28]. The questions were tested internally among a group of Proctor Foundation researchers at the University of California, San Francisco (UCSF), and this group's comments on the question format and phrasing were integrated into the final survey.

After the UCSF Institutional Review Board granted the study's exemption of approval, the survey was disseminated by email via SurveyMonkey (https://www.SurveyMonkey.com/, LLC; Palo Alto, CA). The email message contained a link to the survey and indicated the nature of the survey, that participation was voluntary and that responses would be anonymous. The survey was sent once to the entire group of potential participants and remained open for one week. Recipient responses and inquiries sent directly to the investigators were tracked and categorized.

The response rate was conservatively calculated with the number of completed surveys as the numerator and the total number of surveys sent as the denominator. Participants were able to opt out of responding to any question, and all responses were included in the analysis. For analysis, Likert categories were collapsed into “Agree” and “Disagree,” with “Neutral” responses excluded. Missing data were excluded from analyses.

Results were summarized with descriptive univariate analyses using percentages for categorical data. Bivariate analyses were conducted to compare responses by area of work using Fisher's exact test. The authors examined reliability through a subjective comparison of results to those of other surveys with similar questions. The validity of the survey was examined by asking the tragedy of commons questions in multiple ways and then comparing resulting responses using McNemar's test for paired comparisons. Analyses used Stata statistical software, version 10.0 (StataCorp LP, College Station, TX), or the R program (R Foundation for Statistical Computing, Vienna, Austria). Multiple statistical comparisons were made, so *P* < 0.001 was considered to be significant.

## 3. Results

The literature search identified 16,575 unique email addresses. After removing email addresses that were invalid or that had opted out of receiving email from SurveyMonkey, the survey was sent to 15,508 email addresses. A total of 1,530 responses were received for a response rate of 9.9%. The investigators received 129 direct emails, of which 31 (24.0%) indicated the recipient was out of the office, 22 (17.1%) indicated the recipient was not an infectious disease expert, and 16 (12.4%) indicated the survey could not be completed because the link was already closed. The remaining 60 (46.5%) emails included a variety of comments and questions about the survey itself.

Table 1 displays the characteristics of the survey respondents. The majority of participants had earned either a professional degree (22.9%) or doctoral degree (69.2%). Among those who responded “Other” degree, most indicated multiple degrees, such as M.D./Ph.D. Most worked in microbiology (50.7%), clinical care (24.4%), public health (11.6%), or mathematical modeling of infectious diseases (3.7%). Of those working primarily in clinical care, 92.6% had prescribed antibiotics in the past 5 years.

A summary of responses by area of work is shown in Table 2. Nearly all participants responded that they believed antibiotic resistance is a major health problem (98.6%) and that overuse of antibiotics is a major cause of resistance (97.7%). Respondents also believed that an individual decision to prescribe antibiotics could impact antibiotic resistance in a population (95.3%) and that physicians should weigh the benefits to the patient against the harm to society before prescribing antibiotics (85.8%). A majority of participants believed antibiotic use benefits society (92.6%), but most participants also believed that limiting the use of antibiotics would benefit society (91.2%). Though participants were divided on whether physicians should only consider patient needs when prescribing antibiotics (Table 2), many respondents believed that immediate patient wellbeing is the most important consideration in this decision (79.1%). The vast majority of participants did not believe mass antibiotic treatment programs could produce benefits in either an antibiotic-free society (91.4%) or currently in the United States (94.2%). Of those who believed in some benefit of mass antibiotic distribution, more participants believed that such programs would benefit an antibiotic-free society more than the United States (*P* < 0.001). Participants generally believed that either antibiotic-resistant strains spread more easily in a population (39.7%) or that both resistant and susceptible strains spread equally well (43.0%).

Overall, most responses did not vary significantly by area of work, though a few differences were noted (Table 2). Compared to other areas of work, a greater proportion of those working in clinical care disagreed that physicians should only consider patient needs when prescribing antibiotics (*P* < 0.001) and believed patient wellbeing to be the most important consideration in deciding whether to prescribe antibiotics (*P* < 0.001). Though not significant, more modelers believed that antibiotic-susceptible strains spread more easily than antibiotic-resistant strains (*P* = 0.04 omnibus, *P* = 0.004 comparing modelers versus everyone else). Additionally, a greater proportion of those working in clinical care responded that antibiotic-susceptible strains were more virulent than other groups (*P* = 0.002 omnibus, *P* < 0.001 comparing clinicians versus everyone else).

The majority of participants believed that antibiotic use could result in a tragedy of the commons (Table 2), with 72 (5.1%) responding always, 522 (37.1%) typically, and 758 (53.8%) in certain circumstances. When compared to responses to other questions, this group also believed that the overuse of antibiotics leads to resistance (*P* < 0.001) and that limiting the use of antibiotics would benefit society (*P* < 0.001). These participants also tended to believe that antibiotic resistance is a major public health problem (*P* = 0.007), that an individual decision to use antibiotics impacts population-level resistance (*P* = 0.02), and that physicians should weigh benefits to the individual against harm to society before prescribing (*P* = 0.004). Those who believed antibiotic use would never result in a tragedy of the commons were more likely to believe that physicians should only consider the needs of the patient when prescribing antibiotics (*P* < 0.001) and that antibiotic-susceptible and antibiotic-resistant strains spread equally well in populations (*P* < 0.001). This group also tended to respond that these strains are equally virulent (*P* = 0.003), although the association is not significant.

## 4. Discussion

Many suggest that antibiotic use results in a tragedy of the commons [3–5]. In other words, individual benefits from antibiotic use may lead to overuse and result in a loss of antibiotic effectiveness, to the detriment of the larger society. In practice, however, the balance between individual and societal interests may be more subtle. In some settings, programs to reduce antibiotic use are being implemented, while elsewhere mass antibiotic treatment programs are being proposed [8–10, 16, 17]. Mathematical models have yet to show that a tragedy of the commons is a foregone conclusion; in fact, few published models even allow this scenario, and it may only occur under certain conditions [11–15]. With this survey, we attempted to understand how infectious disease professionals perceive the individual and societal effects of antibiotic use and resistance.

In our survey, most respondents believed such a conflict between individual and societal goals could result from antibiotic use. Multiple questions were used to elicit opinions on this conflict, all of which indicated similar beliefs. Though this survey was not designed to detail the specific requirements for this scenario to occur, it does provide some evidence that infectious disease professionals believe that antibiotic use can lead to a tragedy of the commons. This was seen across disciplines and the majority of respondents appeared to agree with previous models that require specific circumstances for a tragedy of the commons to occur. It is, however, difficult to know whether those who indicated that a tragedy of the commons could occur in “certain circumstances” responded this way because they had specific scenarios in mind or because it was the most neutral response option available. Despite this, 42.2% responded that they believed this could occur “always” or “typically.”

Most of those surveyed here indicated a belief in no benefits from mass treatment for either individuals or society. Yet, previous studies have shown that mass treatment can result in both individual- and community-level benefits [18, 21–26]. In our survey, more respondents believed mass treatment in antibiotic-free societies may produce benefits compared to that in the United States. In fact, proposals are being made to broaden the goals of mass antibiotic treatment to include multiple infectious diseases and even overall mortality in such areas, including a three-country trial to examine the effects of mass antibiotic distribution on mortality [26]. At the same time, mass treatment with antibiotics may increase antibiotic resistance, even in areas with limited previous exposure to antibiotics. For example, longitudinal studies and cluster-randomized trials have shown that community antibiotic distributions result in increased resistance [31–33]. The clinical significance of the emergence of resistance from mass treatment programs has yet to be fully evaluated, but the conflict highlights the need for awareness of both the positive and negative effects of mass treatment in order to properly balance priorities in related policy and programs.

Some variations were noted among responses by area of work. Those working in clinical care were more likely to think that patient well-being is the most important consideration when prescribing antibiotics also that physicians should consider societal effects of antibiotics when making such decisions. These results agree with previous surveys that indicated a high level of physician awareness of the societal consequences of antibiotic resistance along with the practical nature of the importance of individual patient well-being [28].

Our results largely agreed with those of previous surveys that were conducted to assess knowledge, attitudes, and perceptions of physicians on antibiotic use and resistance in clinical settings. These studies also found that physicians believe antibiotic resistance is a significant public health problem and agree antibiotic overuse contributes to its development [27–30, 34–37]. It should be noted that other factors contributing to resistance have also been considered, including the overuse and misuse of antibiotics in agricultural settings [29, 34, 37]; however, this was not a focus of this survey or the other surveys mentioned here. These surveys generally identified a high level of support for antibiotic stewardship programs, though not necessarily for strict restrictions on antibiotic use [30, 37]. Our own survey differs from previous surveys in that it aimed to assess perspectives of not only physicians, but also those working in various infectious disease-related professions. We also included unique questions to evaluate opinions on mass treatment programs.

Strengths of this study include the large, diverse sample and the consideration of reliability and validity in the survey design. The questions were developed in light of previous surveys and used variations of previous survey questions with similar resulting responses. The tragedy of the commons questions were also asked in multiple ways to provide evidence of validity.

This study suffers from several limitations. Though the low response rate was expected from our broad approach, the ability to generalize from these results is necessarily limited. We tracked direct emails from potential participants to provide some inference about those of unknown eligibility and found that a number of recipients were incorrectly contacted or could not respond during the survey period. As we did not collect author names, we were unable to prevent authors from receiving and responding to the survey more than once if they had multiple email addresses. Also, though the majority of publications identified in the literature search were in English, it is possible that language was a barrier to completing the survey for some of the selected authors.

The survey questions and format themselves are also potential limitations. We favored a general approach over a focus on specific antibiotics and infections, and this lack of context may have influenced responses. The broad phrasing of the questions pertaining to mass antibiotic treatment should also be considered when interpreting responses. Responses might have differed if specific scenarios, such as mass azithromycin treatment for the prevention of trachoma in sub-Saharan Africa, had been proposed. The phrasing of other questions may have affected the resulting responses as well. For example, if the question, “Do you believe that antibiotic use results in a tragedy of the commons?,” was worded in a more neutral manner, perhaps there would have been a different distribution of responses. We also focused on clinical settings despite the massive use of antibiotics in agricultural settings. The simplicity of the survey format was not conducive to the expression of complex ideas and this lack of complexity should be considered when interpreting results.

## 5. Conclusions

In conclusion, we found that nearly half of infectious disease experts believed that antibiotic use typically results in a tragedy of the commons. This is somewhat at odds with previous mathematical modeling studies that have only rarely found a tragedy of the commons. Models may not be incorporating the insight of these surveyed experts. On the other hand, a true tragedy of the commons may be more elusive than the experts believe. Future studies could assess the specific circumstances in which antibiotic use could result in a tragedy of the commons, either through transmission models or perhaps even community-randomized studies in the appropriate context.

## Figures and Tables

**Table 1:** Characteristics of participants (*N* = 1530)^1^.

Variable	*N*	%
Highest degree obtained	1430	100.0
Bachelor's or equivalent (B.A., B.S., etc.)	6	0.4
Master's or equivalent (M.A., M.P.H., M.S., etc.)	66	4.6
Professional or equivalent (D.D.S., J.D., M.D., etc.)	328	22.9
Doctoral or equivalent (Ed.D., Ph.D., etc.)	989	69.2
Other^2^	41	2.9
Area of work	1431	100.0
Clinical care	349	24.4
Microbiology	726	50.7
Public health	166	11.6
Mathematical modeling of infectious diseases	53	3.7
Other^3^	137	9.6
Prescribed antibiotics in past 5 years^4^	349	100.0
Yes	323	92.6
Location of work^5^	1493	100.0
Africa	71	4.8
Asia	173	11.6
Australia/New Zealand	42	2.8
Canada/USA	535	35.8
Europe	538	36.0
Latin America	75	5.0
Middle East	54	3.6
Other^6^	5	0.3

^1^Note that participants were allowed to opt out of responding to any question, so the total number of responses varies depending on the particular question.

^2^Participants only allowed one response. “Other” category was open-ended and included responses with multiple degrees such as M.D./Ph.D.

^3^Veterinary medicine, drug development, environmental sciences, and so forth.

^4^Among those who primarily work in clinical care, *N* = 349.

^5^Participants allowed multiple responses.

^6^Eurasion countries such as Turkey, Armenia, and Russia as well as Australasian countries such as Oceania.

**Table 2:** Comparisons by area of work (*N* = 1530)^1^.

Variable	Area of work^2^								Total		*P* value^3^
	Clinical care		Microbiology		Public health		Modeling				
	*N*	%	*N*	%	*N*	%	*N*	%	*N*	%	
Antibiotic resistance is a major public health problem											
Agree	341	98.0	708	98.7	162	99.4	47	97.9	1258	98.6	0.50
Disagree	7	2.0	9	1.3	1	0.6	1	2.1	18	1.4	
The overuse of antibiotics is a major cause of resistance											
Agree	339	98.6	670	97.5	160	98.2	48	92.3	1217	97.7	0.08
Disagree	5	1.5	17	2.5	3	1.8	4	7.7	29	2.3	
An individual decision to use antibiotics has an impact on antibiotic resistance in a population											
Agree	311	96.6	589	94.4	141	95.3	41	97.6	1082	95.2	0.49
Disagree	11	3.4	35	5.6	7	4.7	1	2.4	54	4.8	
Physicians should only consider the needs of the patient when prescribing an antibiotic											
Agree	120	41.4	345	58.9	57	41.6	14	35.9	536	51.0	<0.001
Disagree	170	58.6	241	41.1	80	58.4	25	64.1	516	49.0	
Before prescribing an antibiotic, physicians should weigh the potential benefit to the patient against the potential harm to society											
Agree	260	88.4	504	83.7	130	92.2	33	76.7	927	85.8	0.01
Disagree	34	11.6	98	16.3	11	7.8	10	23.3	153	14.2	
The use of antibiotics benefits society											
Agree	280	91.5	587	92.2	140	95.2	48	98.0	1055	92.6	0.26
Disagree	26	8.5	50	7.9	7	4.8	1	2.0	84	7.4	
Limiting the use of antibiotics would benefit society											
Agree	290	92.4	590	90.2	134	93.1	43	91.5	1057	91.2	0.61
Disagree	24	7.6	64	9.8	10	6.9	4	8.5	102	8.8	
When determining whether or not to prescribe antibiotics, which consideration is more important?											
Long-term societal wellbeing	40	11.7	174	24.8	40	24.2	9	18.0	263	20.9	<0.001
Immediate patient wellbeing	301	88.3	529	75.3	125	75.8	41	82.0	996	79.1	
In an antibiotic-free society, would it be beneficial to provide everyone with a broad-spectrum antibiotic nonspecifically once per year?											
Yes^4^	19	5.6	65	9.2	18	11.2	6	11.8	108	8.6	0.08
No	321	94.4	644	90.8	143	88.8	45	88.2	1153	91.4	
In the United States in 2012, would it be beneficial to provide everyone with a broad-spectrum antibiotic nonspecifically once per year?											
Yes^4^	11	3.3	49	7.2	9	5.6	3	6.0	72	5.8	0.07
No	327	96.8	632	92.8	153	94.4	47	94.0	1159	94.2	
Do you think that antibiotic use results in a “tragedy of the commons”?^5^											
Yes (always or typically)	154	44.8	283	40.5	77	47.2	21	40.4	535	42.5	0.57
Under certain circumstances	179	52.0	387	55.4	79	48.5	28	53.9	673	53.5	
No	11	3.2	29	4.2	7	4.3	3	5.8	50	4.0	
In general, which type of strain will spread more easily in a population?											
Antibiotic-susceptible	64	18.7	107	15.3	28	17.8	17	35.4	216	17.3	0.043
Antibiotic-resistant	131	38.3	289	41.3	62	39.5	12	25.0	494	39.7	
Neither	147	43.0	303	43.4	67	42.7	19	39.6	536	43.0	
In general, which type of strain will be more virulent in an individual?											
Antibiotic-susceptible	74	21.5	85	12.1	17	10.8	4	8.3	180	14.4	0.003
Antibiotic-resistant	101	29.4	259	37.0	59	37.6	19	39.6	438	35.1	
Neither	169	49.1	356	50.9	81	51.6	25	52.1	631	50.5	

^1^Note that participants were allowed to opt out of responding to any question, so the total number of responses varies depending on the particular question.

^2^“Other” category excluded.

^3^<0.001 considered significant.

^4^“Yes” includes collapsed categories.

^5^Question prompt in the survey included the following definition of “tragedy of the commons”: Garrett Hardin's classic dilemma, the “tragedy of the commons,” arises when multiple individuals, acting solely out of their own self-interest, ultimately exhaust a limited shared resource despite the fact that it is in no one's long-term interest for this to happen. A well-known example of this situation involves a group of individuals grazing cattle on a tract of common land. Each individual will seek to maximize his/her gains, and each individual will benefit from grazing additional cattle on the land.

This continued increased use will eventually deplete or even destroy the utility of the common land, and these negative effects will be felt by everyone. Since the negative effects of grazing additional cattle are shared by the group, but the benefits of additional cattle reside with the individual, each rational individual will continue to graze additional cattle despite the ultimate detriment to the group.
